# The Story of SPATA2 (Spermatogenesis-Associated Protein 2): From Sertoli Cells to Pancreatic Beta-Cells

**DOI:** 10.2174/138920209788920976

**Published:** 2009-08

**Authors:** Claudio Maran, Evelyne Tassone, Valentina Masola, Maurizio Onisto

**Affiliations:** Department of Experimental Biomedical Sciences, University of Padova, Italy

**Keywords:** Spata2, Sertoli cells, pancreatic β-cells, microRNAs.

## Abstract

In an attempt to isolate new spermatogenesis-associated genes, pd1 was initially identified and cloned as a novel human cDNA sequence from testis cDNA library. The novel gene was submitted to GenBank under accession n° U28164 in 1996. PD1 expression was demonstrated at the Sertoli cell level with a production which appeared to be under the influence of neighbouring spermatogenic cells. The rat orthologue of human pd1 was further cloned and, according to the Gene Nomenclature Committee, was renamed *spata2 *(spermatogenesis-associated protein 2) gene on the basis of its FSH-dependent up-regulation and developmental expression. The analysis of the human and rat cDNA sequences disclosed an open reading frame for a protein of 520 and 511 amino acids respectively, with an overall identity of 85%. Subsequently, a zebrafish orthologue of the human spata2 gene was identified. The consensus open reading frame (1650 bp) encodes a polypeptide of 550 amino acids, which shares 37% identity with the human spata2. By means of whole-mount *in situ *hybridisation it has been shown that *spata2 *transcripts are maternally derived and become strongly localised in the central nervous system at early developmental stages. At the same time, RT-PCR analysis demonstrated that several adult zebrafish tissues expressed high level of *spata2 *mRNA providing evidence that this gene may have a broader function than previously described. More recently, novel findings have highlighted a potential role of *spata2 *during pancreatic development and β-cell proliferation. In this review we will discuss *spata2 *gene expression and regulation as well as focus on novel evidence, which suggests a role for this protein in pancreatic β-cell function.

## THE BEGINNING OF THE STORY: PD1 AND SERTOLI CELLS

More than ten years ago our laboratory became involved in the effort to isolate new spermatogenesis-associate genes. During a screening of a human testis cDNA library, we isolated and cloned a 1.4 kb cDNA sequence by means of a RACE-PCR strategy. This sequence turned out to be novel and thus was named pd1 and submitted to the GenBank under accession n° U28164 in 1996. Afterwards, a 5’ RACE-PCR was performed in order to identify the real 5’-end of the cDNA and in this way the sequence was extended to 2.6 kb. The sequence analysis revealed an ORF of 1560 bp encoding a 520 amino acid protein with a predicted molecular weight of 58.4 kDa [1]. A Northern blot analysis performed on a multiple human tissue mRNA blot showed that the pd1 transcript is moderately expressed and that the testis is the tissue with highest expression whereas other tissues (spleen, prostate and thymus) have lower level of pd1 expression. To complete the expression analysis, we raised and purified polyclonal antibodies against a recombinant PD1 fragment corresponding to 148 C-terminus amino acids. The immunohistochemical analysis on human normal testis showed an intense staining of the seminiferous tubules with a clear localisation to the cytoplasm of the Sertoli cells whereas no reactivity was observed in any spermatogenic cells at any maturative level. We then performed a Western-blot analysis on crude extract of human testicular cells obtained from fine needle aspiration and corresponding various testiculopathies. This study recognised a band of the expected size (approximately 60 kDa) and showed a reduction of PD1 expression in protein samples retrieved from patients with hypospermatogenesis (sperm count lower than 5 x 10^6^/ml) and a further reduction in the sample from a subject with Sertoli cell-only syndrome (complete absence of germ cell). Therefore, PD1 expression by the Sertoli cells was not constitutive but appeared to be under the influence of neighbouring spermatogenic cells. These observations were recently confirmed by O’Shaughnessy *et al*. who studied the effect of germ cell depletion on the levels of specific mRNA transcript in mouse Sertoli cells and Leydig cells. In this animal model the authors demonstrated that out of 26 Sertoli cell markers tested, *pd1* (currently named *spata2*) was one of the two genes whose expression decreased as germ cell depletion progressed [2]. It has been observed that a similar behaviour was characteristic of inhibin B which is a protein secreted by Sertoli cells, involved in the feedback regulation of FSH secretion and whose expression reflects interactions between these cells and neighbouring germ cells [3, 4]. Although there was not any homology between pd1 and inhibin B, the similar expression pattern with respect to germ cells prompted us to hypothesise that PD1 might have a role in the regulation of human spermatogenesis [1].

## THE RAT ORTHOLOGUE OF HUMAN PD1: *SPATA2* (SPERMATOGENESIS-ASSOCIATED PROTEIN 2)

With the aim of further elucidating the role of pd1 on the control of spermatogenesis, we decided to isolate the rat orthologue of this gene. Using a set of primers designed on the basis of human pd1 gene, the entire coding sequence was cloned from a rat testis cDNA pool and subsequently completed with 5’ and 3’ UTR regions [5]. Sequence analysis revealed an open reading frame encoding a protein of 511 amino acids with an overall identity of 85% between human and rat proteins. Based on the fact that the basic mechanisms underlying the process of sexual development are conserved across species and since FSH is the most important molecule regulating Sertoli cells function, time-course experiments were performed on rat Sertoli cells to study *pd1* mRNA expression following FSH stimulation. This treatment induced, in a time-dependent manner, a remarkable increase in *pd1* mRNA level demonstrating that the expression of this protein is FSH-responsive. In the same report the expression of *pd1* mRNA in rat testis during testicular development has been analysed and clear evidences that *pd1* transcripts appeared during the infantile-juvenile period and increased steadily with advancement of age were provided. On the whole, these results confirmed the hypothesis that PD1 might play a role in regulating spermatogenesis and, thus, according to the Gene Nomenclature Committee, the name SPATA2 (Spermatogenesis-associated protein 2) has been proposed for this protein.

## THE GENOMIC ORGANIZATION AND EXPRESSION PATTERN OF SPATA2 GENE

To comprehend the transcriptional control and the genomic organization of *spata2* gene, we decided to characterise its promoter region and the functional subregion that control its expression. Once the exact position of the transcription start point was located, the genomic organisation of *spata2* gene was determined by means of BLASTN alignment with genomic sequence deposited in GenBank. In this way, we showed that the genomic organisation of *spata2* gene is composed of three exons and two introns spanning a region of about 12.1 kb and confirmed the chromosome localisation at 20q13.13 [6].

A promoter region analysis was also performed which revealed the presence of potential binding sites for various transcription factors as well as the absence of a canonical TATA box. In addition, using a luciferase gene-reporter assay it has been demonstrated that the basal promoter activity is driven by Sp1 elements near the transcriptional start site whereas the strongest promoter activity is determined by the -403/+159 region containing a CREB site.

It is noteworthy that sequence similarity searches for spata2 do not reveal a significant homology to any other gene or protein. At the same time, bioinformatic analyses reveal a small pattern PW [KR] KE [YF][RK], which appears to be of particular interest due to its strong conservation between SPATA2 and the *D. melanogaster* TAMO protein. TAMO has been demonstrated to be a protein involved in nuclear import machinery during oogenesis in *D. melanogaster* [7].

Subsequently, with the goal of getting new insights into SPATA2 function, we decided to identify a potential orthologue in zebrafish (*Danio rerio*). Thanks to its transparency, at early developmental stages together with its easy manipulation and high reproductive capability, this animal has become extensively used as a genetic tool to uncover specific functions of unknown proteins as well as to study human disease [8, 9]. Starting from a zebrafish cDNA library, we cloned and characterised a 2905 bp cDNA sequence which represents the zebrafish orthologue of the human *spata2* gene and whose consensus open reading frame (1650 bp) encodes a polypeptide of 550 amino acids sharing a 37% identity with the human protein [10]. Whole-mount *in situ* hybridization detected spata2 transcript at two-cell stage (thus demonstrating a maternal origin of the transcript), whereas at early developmental stages a strong signal was ubiquitously visible and became localised in the central nervous system by 48 h post fertilisation. To address the question as to whether zebrafish spata2 maintains its ubiquitous spatial expression in adult stages, a multi tissue RT-PCR analysis was performed and it has been demonstrated that almost all analysed tissues of adult fish do display a high content of *spata2 *transcripts. A great amount of data, available on public domain databases (GEO Profile, Gensat) confirm this observation highlighting once more that spata2 has a wide distribution in several normal and pathological tissues and that, among them, testis and brain display the highest expression levels.

In conclusion, these different analyses performed in zebrafish, rodents and human tissues, provided evidence that spata2 may have a broader function than previously described.

## THE LAST PART OF THE STORY: SPATA2, MIR-7 AND PANCREATIC ISLETS

MicroRNAs (miRNAs) are a novel class of short non-coding RNAs that regulate gene expression at post-transcriptional level. They generally bind to the 3’-UTR of their target mRNAs, thus inhibiting mRNAs translation [11, 12]. miRNAs expression is often tissue- or developmental-specific and plays an important role in the regulation of gene expression at specific stages in various biological processes such as in the case of the differentiation of endocrine pancreas [13, 14]. Human pancreas develop from the ventral and dorsal buds of foregut endoderm and its efficient development needs spatio-temporal changes in gene expression too. Recent data demonstrate the expression of specific miRNAs in pancreas and suggest a direct role for miRNAs in mammalian insulin secretion [15], as well as during the development of pancreatic islets in zebrafish [16]. Bravo-Egana *et al*. performed a differential expression analysis of miRNAs in human adult acinar and islets pancreatic tissue and identified miR-7 to be the most highly and selectively expressed miRNA in islets, with an islet/acinar ratio of expression greater than 200 [17]. Other studies confirmed the major expression of miR-7 during human pancreatic islet development and differentiation and revealed the correlation between its expression and the observed increase in pro-insulin gene transcripts [18, 19]. The RNA target predictive algorithms TargetScan, Miranda and PicTar identified spata2 gene as the miR-7 target with the highest rank. Moreover, miR-7 silencing experiments, performed with anti-miR-7 morpholinos, demonstrate an increase in the expression of spata2 mRNA (in *ex vivo* cultured islets or in the pancreatic β-cell line MIN6) thus confirming that spata2 is down-regulated by miR-7. Since spata2 and miR-7 transcript expression seems to be inversely correlated, it has been proposed that spata2/miR-7 could be involved in islet biogenesis [20].

In any case, a possible link between SPATA2 and pancreatic islets was first suggested some years ago by Luca *et al*., who demonstrated that *in vitro* co-incubation of rat pre-pubertal Sertoli cells, or their secreted products, with homologous islets resulted in statistically significant increase of the islet β-cell mitotic index. Using polyclonal antibodies against-PD1/SPATA2 provided by our laboratory, they showed an evident decline of the Sertoli cells-related pro-mitogenic effects on the β-cells and hence these authors hypothesised that PD1/SPATA2 might be one of the soluble factors involved in the induction of β-cell mitotic activity [21]. Interestingly, preliminary immuno-chemical results from our laboratory seem to confirm an endogenous production of spata2 by human pancreatic β-cells since: 1) in human normal pancreas sections, an evident positive stain for this protein co-localised with that of insulin, which is a marker of these endocrine cells; 2) in MIN6 β-cell line [22], an RT-PCR analysis reveals the presence of spata2 transcripts and an ELISA assay detects the presence of the relative protein in their conditioned media respectively.

On the whole, these pieces of evidence suggest that SPATA2 might be produced and secreted by β-cells and could act as a autocrine/paracrine molecule able to induce a pancreatic islets mitogenesis.

Whether or not this hypothesis will be confirmed, spata2 may represent a useful tool in addressing the growing request of new therapeutic strategies based on β-cells renewal and aimed at overcoming the well-known phenomenon of β-cells destruction typical of *Diabetes mellitus.* In this regard, many studies are actually targeted at understanding and identifying those factors that regulate β-cells proliferation and function [23].

The demonstration that spata2 turned out to be a “growth factor” for β-cells is awaiting the results of further investigations which are currently in progress in our laboratory.
